# Dependence symptoms and cessation intentions among US adult daily cigarette, cigar, and e-cigarette users, 2012-2013

**DOI:** 10.1186/s12889-016-3510-2

**Published:** 2016-08-18

**Authors:** Brian L. Rostron, Megan J. Schroeder, Bridget K. Ambrose

**Affiliations:** Center for Tobacco Products, Food and Drug Administration, 10903 New Hampshire Avenue, Silver Spring, MD 20993 USA

**Keywords:** Tobacco, Dependence, Cessation, Cigarettes, Cigars, E-cigarettes

## Abstract

**Background:**

Cigar and e-cigarette use is becoming increasingly common among US tobacco users and the Food and Drug Administration recently asserted regulatory jurisdiction over these products, among others, in May 2016. Research on tobacco dependence among users of these products is limited, however. We therefore examined several symptoms of dependence and cessation intentions among adult cigarette, cigar, and/or e-cigarette users in a nationally representative sample.

**Methods:**

We used nationally representative data from more than 60,000 participants in the US National Adult Tobacco Survey (NATS) from 2012 to 2013 to analyze dependence symptoms and cessation intentions for users of cigarettes, cigars, and/or e-cigarettes but not other tobacco products.

**Results:**

Among daily tobacco users, dual cigarette and cigar users on average smoked more cigarettes per day (17.3, 95 % CI = 16.1, 18.6 vs. 15.8, 95 % CI = 15.4, 16.2), had shorter times to first tobacco use after waking (21.4 min, 95 % CI = 16.6, 24.9 vs. 25.9 min, 95 % CI = 25.3, 26.5), and were more likely to report withdrawal and craving symptoms than exclusive cigarette smokers. Dual cigarette and e-cigarette users were more likely than exclusive cigarette smokers to report withdrawal and craving symptoms and cessation intentions. Exclusive cigar and e-cigarette users were less likely to report withdrawal and craving symptoms than users of other products, but even so, more than a third of exclusive cigar (38.8 %, 95 % CI = 27.4 %, 51.6 %) and e-cigarette (46.1 %, 95 % CI = 35.1 %, 57.4 %) users reported experiencing a strong craving for a tobacco product in the past 30 days.

**Conclusions:**

Dual cigarette and cigar users show evidence of greater dependence symptoms and dual cigarette and e-cigarette users show evidence of greater dependence symptoms and cessation intentions compared with exclusive cigarette smokers. A sizeable number of users of all of the tobacco products report dependence symptoms such as craving for tobacco.

## Background

Tobacco users in the United States (US) are increasingly likely to use products other than or in addition to cigarettes [1–3], including some products newly regulated by the US Food and Drug Administration (FDA) in May 2016 such as cigars and e-cigarettes, but nationally representative data on dependence and cessation intentions among users of these products are limited. Previous research on tobacco dependence has primarily focused on cigarette smokers, and the majority of dependence measures assess dependence based on questions related to cigarettes. For example, the Fagerström Test for Cigarette Dependence (previously the Fagerström Test for Nicotine Dependence [4, 5], has been used extensively to assess nicotine dependence among cigarette smokers [6–9]. Several studies have found that two of the test’s items, cigarettes per day (cpd) and time to first cigarette after waking (ttfc), are highly predictive symptoms of dependence [10–12], and these two elements comprise the commonly used Heaviness of Smoking Index [10]. Other cigarette dependence scales, such as the Wisconsin Index of Smoking Dependence Motives [13], ask about dependence symptoms such as craving and tolerance.

Measures and studies of dependence among users of other tobacco products such as cigars and e-cigarettes are much more limited, even though non-cigarette tobacco products contain nicotine and users show signs of dependence [14]. Researchers have used National Youth Tobacco Survey data to analyze the prevalence of dependence symptoms such as feeling restless or irritable after not using tobacco for a while in cigarette, cigar, and smokeless tobacco users among middle and high school students [15]. Researchers have also used methods from item response theory with National Epidemiological Survey of Alcohol and Related Conditions data to develop measures of nicotine dependence among users of cigarettes, cigars, and other tobacco products [16]. The Penn State Electronic Cigarette Dependence Index has also been proposed to assess dependence in exclusive e-cigarette users [17].

Even with these efforts, information from nationally representative studies about tobacco dependence among users of products such as cigars and e-cigarettes remains limited. The FDA issued a federal rule in May 2016 asserting regulatory jurisdiction over all tobacco products in the US that were not previously regulated, including e-cigarettes and cigars [18]. Understanding dependence symptoms and cessation intentions among users of these products is therefore an important public health issue, and results from this study will help provide information about their effects on users that can inform future regulatory and research activities.

Our study addresseses these issues by using nationally representative data for US adults from the 2012–2013 National Adult Tobacco Survey (NATS) to analyze the prevalence of tobacco dependence symptoms among exclusive cigarette, cigar, and e-cigarette users, as well as users of combinations of these products. We restricted our analyses to these three tobacco products because they are associated with similar use behaviors, including possible inhalation and hand and mouth interaction, thus allowing for more direct comparison for some dependence symptoms. The 2012–2013 NATS was designed to include multiple measures of tobacco dependence symptoms relevant for all tobacco products, including craving and withdrawal symptoms and time to first tobacco use. Because tobacco dependence is often predictive of cessation motivation and success [11, 16], our study also analyzes future cessation intentions for users. This study provides valuable insight into tobacco dependence and cessation intentions among US adults, particularly for cigar and e-cigarette users for whom such information was previously lacking.

## Methods

### Data source

The 2012–2013 NATS is a stratified, random-digit dialed, landline and cellular telephone survey of US non-institutionalized adults aged 18 years and older residing in the 50 states and the District of Columbia [19]. Data collection occurred from October 1, 2012 through July 30, 2013, with 57,994 completed interviews and 2,198 eligible partial interviews (≥60 % complete), yielding 60,192 total qualified interviews [20]. NATS utilized a dual frame, non-overlapping sample design, with independent samples drawn from landline and cellular-only telephone frames. The cellular-only frame consisted of households without landlines that rely exclusively on cellular telephones to make calls.

### Measures

#### Tobacco product use

The 2012–2013 NATS assessed use of cigarettes, cigars (including large cigars, cigarillos, and little filtered cigars), and e-cigarettes, among other tobacco products including traditional pipes, waterpipes/hookah, chewing tobacco, snuff, dip, snus, and dissolvable tobacco products. Our analysis focused on daily cigarette, cigar, and/or e-cigarette users although descriptive statistics for all daily tobacco users are presented for purposes of comparison. Current cigarette and cigar smokers aged 30 years and older were defined as individuals reporting having smoked at least 100 cigarettes or 50 cigars in their lifetimes and currently using these products every day or some days for cigarettes and every day, some days, or rarely for cigars. Young adults aged 18–29 years reporting current daily or some day use of cigarettes or daily, some day or rare use of cigars did not need to meet the thresholds for ever product use to be classified as a current user, but most daily cigarette smokers (6,066 of 6,073) and daily cigar smokers (339 of 340) reported that they had met the relevant threshold. Current e-cigarette users reported using these products every day, some days, or rarely. Tobacco users analyzed in this study were categorized as exclusive users of cigarettes, cigars, and e-cigarettes; dual users of cigarettes and cigars or cigarettes and e-cigarettes; or users of all three of these products. Users of these products along with other tobacco products were excluded from the analysis. Exclusive daily use of traditional pipes or waterpipes was extremely low among NATS participants, with 31 and 1 users respectively.

#### Daily use

Survey participants were identified as daily users for this analysis in one of two ways. First, respondents could report every day use of cigarettes, cigars, and/or e-cigarettes. Second, respondents could report some day use of at least two of these products and that there were not days when they did not use any of these products.

#### Symptoms of tobacco dependence

NATS included several questions to assess tobacco dependence symptoms, including average number of cigarettes smoked per day, time to first tobacco use after waking, and questions about withdrawal and craving symptoms. These questions included whether or not respondents sometimes wake at night to use a tobacco product, have had a strong craving to use any tobacco product in the past 30 days, have felt like they really needed to use a tobacco product in the past 30 days, and have had a time when they wanted to use a tobacco product so much that it was difficult to think of anything else in the past 30 days. Respondents were also asked if the statement that they feel restless or irritable after not using tobacco for a while was “not at all true,” “sometimes true,” “often true,” or “always true.” These dependence measures have been shown to be reliable among adult tobacco users [11, 21].

#### Tobacco use cessation intentions

NATS participants who reported being current cigarette smokers were asked if they were thinking about quitting cigarettes for good, and respondents who answered “yes” were then asked how soon they were likely to quit cigarettes for good with response options being “within the next 30 days,” “within the next 6 months,” “within the year,” “longer than a year,” and “don’t know/not sure.” We calculated the proportion of these respondents who were in one of the first three response groups and thus reported being likely to quit cigarettes within the next year. Cigarette smokers who used other tobacco products and reported they were thinking about quitting cigarettes for good as well as exclusive users of non-cigarette tobacco products were asked if they were thinking of quitting all tobacco products for good. Respondents who reported “yes” were then asked how soon they were likely to quit all tobacco products with the same response options as above. In our analysis, we classified dual and polyusers of both cigarettes and other tobacco products who reported thinking about quitting cigarettes and all tobacco products as thinking about quitting all tobacco products.

#### Demographic characteristics

We analyzed tobacco product use by several demographic characteristics including sex, age (18–29, 30–50, and > 50 years), race/ethnicity (non-Hispanic white, non-Hispanic African-American, non-Hispanic other or multi-race, and Hispanic), and educational attainment (less than high school diploma or equivalency degree, high school diploma or equivalent, some post high school or college course work or degree but not a bachelor’s degree, and a bachelor’s degree and/or master’s, professional, or doctoral degree). Respondents with missing or incomplete information about tobacco use, dependence symptoms, cessation intentions, or demographic characteristics were not included in those particular analyses, unless otherwise noted.

#### Participants

There were a total of 60,192 participants in the 2012–2013 NATS. Of these individuals, 59,640 provided information about current use of all of the surveyed tobacco products, and were thus eligible for further analysis in this study, and 11,654 of these respondents reporting current use of at least one tobacco product. To facilitate comparisons across product use categories, our analysis focused on daily tobacco users, who either reported using a single product type every day or being a multi-product user and using at least one tobacco product every day. Among current users, 7,239 individuals were daily users of any tobacco products, and 5,617 of these were users of some combination of cigarettes, cigars, and e-cigarettes but not other products.

### Statistical methods

Analyses consisted of calculation of proportions of survey responses, mean number of cigarettes smoked per day, and median time to first tobacco use after waking, and accompanying 95 % confidence intervals. Comparisons of means and proportions between product use groups were made using t-tests with *p* < 0.05 as the level of statistical significance. All estimates were calculated using the NATS national sample weights and taking into account the complex survey design stratification information. All analyses were conducted using SAS version 9.4 [22] and SUDAAN version 11.0.1 [23].

## Results

Table 1 presents a detailed breakdown of NATS participants and daily tobacco users by product use category and demographic characteristics including sex, age, race/ethnicity, and educational attainment. A majority (60.9 %, 95 % CI = 59.7 %, 62.1 %) of current tobacco users were daily users with the largest number of these users being daily exclusive cigarette users (31.3 %, 95 % CI = 30.2 %, 32.5 %), dual cigarette and e-cigarette users (6.1 %, 95 % CI = 5.6 %, 6.7 %), and dual cigarette and cigar users (4.6 %, 95 % CI = 4.1 %, 5.1 %). Among daily tobacco users, exclusive cigar smokers were overwhelmingly male (83.8 %, 95 % CI = 72.1 %, 91.2 %), whereas dual cigarette and e-cigarette users were more often female (61.4 %, 95 % CI = 56.5 %, 66.1 %). The product use categories with the largest proportion of young adult users aged 18–29 years were polyusers of cigarettes, cigars, and e-cigarettes (49.4 %, 95 % CI = 38.0 %, 60.9 %) and dual users of cigarettes and cigars (25.0 %, 95 % CI = 20.0 %, 30.7 %). Compared with other product type users, a relatively large proportion of exclusive cigar smokers were non-Hispanic African-Americans (32.4 %, 95 % CI = 20.8 %, 46.6 %). Similarly, a relatively small proportion of exclusive e-cigarette users had less than a high school diploma (3.9 %, 95 % CI = 1.6 %, 9.5 %). Some additional information from NATS on tobacco use by other demographic characteristics such as sexual orientation and household information has been published previously by FDA authors [24].

**Table 1:** Characteristics of National Adult Tobacco Survey Participants by Tobacco Product Use Status (%)

	Proportion of Current Tobacco Users	Sex		Age (years)			Race/Ethnicity			Educational attainment			
		Male	Female	18–29	30–50	>50	Non-Hispanic White	Non-Hispanic African-American	Hispanic	< High School Diploma or Equivalent	HS Diploma or Equivalent	Some Post HS Education, No Bachelor’s Degree	Bachelor's Degree or More
All respondents (*n* = 60192)		48.4 [47.8, 48.9]	51.6 [51.1, 52.2]	21.6 [21.1, 22.2]	36.3 [35.7, 36.8]	42.1 [41.6, 42.6]	65.6 [65.1, 66.2]	10.2 [9.8, 10.6]	15.0 [14.5, 15.5]	13.7 [13.3, 14.3]	28.1 [27.6, 28.7]	31.6 [31.1, 32.1]	26.5 [26.1, 26.9]
Daily any tobacco product users^a^(*n* = 7239)	60.9 [59.7, 62.1]	60.8 [59.3, 62.4]	39.2 [37.6, 40.7]	23.4 [22.0, 24.9]	43.5 [42.0, 45.1]	33.1 [31.7, 34.4]	69.2 [67.7, 70.8]	9.9 [8.9, 11.0]	9.3 [8.3, 10.4]	19.5 [18.1, 21.1]	37.3 [35.8, 38.9]	34.0 [32.5, 35.4]	9.2 [8.5, 9.9]
Daily single tobacco product users													
Cigarettes only (*n* = 3963)	31.3 [30.2, 32.4]	50.1 [47.9, 52.3]	49.9 [47.7, 52.1]	13.4 [11.8, 15.3]	45.1 [42.9, 47.3]	41.5 [39.5, 43.5]	67.4 [65.2, 69.6]	12.4 [10.9, 14.1]	10.0 [8.6, 11.5]	22.3 [20.2, 24.6]	36.4 [34.3, 38.6]	32.2 [30.3, 34.2]	9.0 [8.2, 10.0]
Cigars only (*n* = 131)	1.0 [0.8, 1.3]	83.8 [72.1, 91.2]	16.2 [8.8, 27.9]	19.7 [10.8, 33.2]	36.1 [24.4, 49.8]	44.2 [33.0, 56.1]	49.5 [36.8, 62.2]	34.8 [22.6, 49.5]	^b^	^b^	38.0 [26.0, 51.6]	28.7 [19.5, 40.1]	13.4 [8.4, 20.9]
E-cigarettes only (*n* = 124)	1.1 [0.9, 1.4]	57.2 [45.7, 68.1]	42.8 [31.9, 54.3]	19.3 [11.1, 31.5]	48.8 [37.7, 59.9]	31.9 [23.4, 41.9]	80.1 [69.6, 87.6]	^b^	^b^	^b^	37.8 [27.2, 49.7]	45.8 [34.6, 57.4]	12.5 [7.7, 19.6]
Daily multiple tobacco product users													
Cigarettes + cigars (*n* = 495)	4.6 [4.1, 5.1]	73.6 [68.2, 78.4]	26.4 [21.6, 31.8]	25.0 [20.0, 30.7]	45.9 [40.2, 51.7]	29.1 [24.7, 33.9]	59.8 [53.7, 65.6]	19.1 [14.7, 24.5]	8.0 [5.2, 12.2]	19.3 [14.6, 25.1]	40.2 [34.5, 46.2]	34.2 [28.9, 39.9]	6.3 [4.6, 8.6]
Cigarettes + e-cigarettes (*n* = 774)	6.1 [5.6, 6.7]	38.6 [33.9, 43.5]	61.4 [56.5, 66.1]	15.9 [12.5, 20.1]	48.3 [43.6, 53.1]	35.8 [31.7, 40.0]	76.2 [71.5, 80.3]	5.2 [3.4, 7.9]	7.3 [4.8, 11.1]	14.8 [11.0, 19.6]	35.7 [31.2, 40.5]	39.7 [35.2, 44.4]	9.8 [7.9, 12.1]
Cigarettes + cigars + e-cigarettes (*n* = 130)	1.3 [1.1, 1.7]	64.9 [52.8, 75.3]	35.1 [24.7, 47.2]	49.4 [38.0, 60.9]	34.5 [24.6, 46.0]	16.0 [10.4, 24.0]	57.0 [44.9, 68.3]	^b^	18.8 [10.7, 30.9]	21.1 [12.3, 33.7]	29.2 [19.4, 41.4]	44.9 [33.9, 56.3]	^b^

^a^ Daily users reported either daily use of at least one tobacco product or reported some day use of multiple products and replied “no” when asked if there were some days when they do not use any tobacco products

^b^ relative standard error > 30 %

**Fig. 1:**
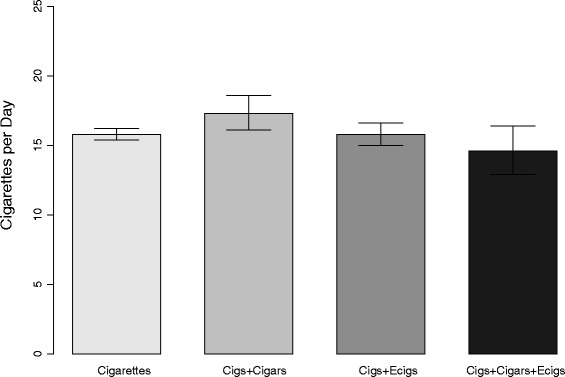
Mean Cigarettes Smoked Per Day Among Daily Tobacco Users. Errors bars represent 95 % confidence intervals

**Fig. 2:**
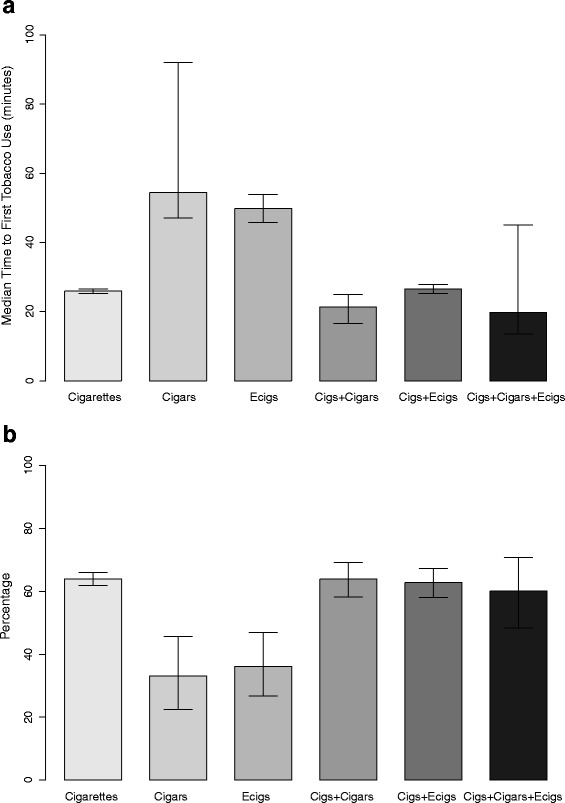
**a** Median Time to First Tobacco Use after Waking among Daily Tobacco Users. **b** Proportion of Daily Tobacco Users Using a Tobacco Product Within 30 min of Waking. Errors bars represent 95 % confidence intervals

Figure 1 shows the mean number of cigarettes smoked per day (cpd) by daily cigarette smokers in the various product use categories. Mean cpd for dual cigarette and cigar smokers was higher at 17.3 than for exclusive cigarette smokers at 15.8 (*p* = 0.02). Mean cpd for dual cigarette and e-cigarette users and cigarette, cigar, and e-cigarette polyusers were not different from that of exclusive cigarette smokers. Dual cigarette and cigar smokers also had a shorter median time to first tobacco use after waking (21.4 min, 95 % CI = 16.6, 24.9) than exclusive cigarette smokers (25.9 min, 95 % CI = 25.3, 26.5), as shown in Fig. 2a. Exclusive cigar and e-cigarette users had a longer median time to first use than exclusive cigarette smokers. Exclusive cigar and e-cigarette users were also less likely to use these products within 30 min of waking compared with exclusive cigarette smokers (both *p* < 0.0001), as shown in Fig. 2b.

Other tobacco dependence symptoms are analyzed in Table 2. Dual and polytobacco users were more likely than exclusive cigarette smokers to demonstrate several dependence symptoms. For example, dual cigarette and cigar users were more likely than exclusive cigarette smokers to awaken at night to use tobacco products (*p* = 0.002). Dual cigarette and cigar users, dual cigarette and e-cigarette users, and cigarette, cigar, and e-cigarette polyusers were also more likely than exclusive cigarette smokers to report a strong craving for tobacco in the past 30 days (*p* = 0.001, *p* < 0.0001, and *p* = 0.001). Dual cigarette and e-cigarette users and cigarette, cigar, and e-cigarette polyusers were more likely to report that they felt like they really needed to use tobacco in the past 30 days than exclusive cigarette smokers (*p* < 0.0001 and *p* = 0.02). Dual cigarette and cigar users as well as dual cigarette and e-cigarette users were more likely than exclusive cigarette smokers to report wanting to use a tobacco product so much that it was difficult to think about anything else during the past 30 days (*p* = 0.02 and *p* = 0.002). These dual users were also more likely than exclusive cigarette smokers to report often or always feeling restless or irritable after not using tobacco for a while. Even though exclusive cigar and e-cigarette users were less likely than users of other products to report withdrawal and craving symptoms, many of these users still reported dependence symptoms (*p* = 0.02 and *p* = 0.003). For example, more than a third of exclusive cigar (38.8 %, 95 % CI = 27.4 %, 51.6 %) and e-cigarette (46.1 %, 95 % CI = 35.1 %, 57.4 %) users reported experiencing strong cravings for tobacco in the past 30 days. Similar proportions of exclusive cigar (40.3 %, 95 % CI = 28.4 %, 53.5 %) and e-cigarette (46.2 %, 95 % CI = 35.2 %, 57.5 %) users also reported feeling like they really needed to use tobacco in past 30 days.

**Table 2:** Prevalence of Tobacco Dependence Symptoms Among Daily Tobacco Users (%)

	Sometimes wake up at night to use a cigarette or other tobacco product	Have had a strong craving to use a tobacco product in past 30 days	Ever felt like they really needed to use a tobacco product in past 30 days	Wanted to use a tobacco product so much that they found it difficult to think of anything else in past 30 days	Feel restless or irritable after not using tobacco for a while, often or always true^a^
Single Tobacco Product Use					
Cigarettes only	21.6 [19.8, 23.5]	61.8 [59.6, 63.9]	66.9 [64.8, 69.0]	19.8 [18.1, 21.7]	41.7 [39.6, 43.9]
Cigars only	^b^	38.8 [27.4, 51.6]*	40.3 [28.4, 53.5]*	^b^	^b^
E-cigarettes only	^b^	46.2 [35.2, 57.5]*	46.2 [35.2, 57.5]*	^b^	22.8 [14.8, 33.4]*
Multiple Tobacco Product Use					
Cigarettes + cigars	30.6 [25.4, 36.2]*	70.9 [65.5, 75.8]*	71.3 [65.9, 76.1]	26.4 [21.5, 32.0]*	49.4 [43.5, 55.2]*
Cigarettes + e-cigarettes	22.7 [18.8, 27.1]	77.1 [73.0, 80.8]*	80.6 [76.9, 83.9]*	27.3 [23.2, 31.8]*	49.7 [45.0, 54.5]*
Cigarettes + cigars + e-cigarettes	30.2 [20.0, 42.9]	77.4 [67.2, 85.1]*	78.1 [67.7, 85.8]*	21.8 [14.0, 32.4]	39.1 [28.4, 50.9]

* *p* < 0.05 compared with cigarette only users

^a^ Respondents were asked if the statement “After not using tobacco for a while, I feel restless or irritable” was “not at all true,” “sometimes true,” “often true,” or “always true.”

^b^ relative standard error > 30 %

Table 3 presents results for cessation intentions. Dual cigarette and e-cigarette users were more likely (87.4 %, 95 % CI = 84.0 %, 90.2 %) than exclusive cigarette smokers (75.6 %, 95 % CI = 73.7 %, 77.5 %) to report thinking about quitting cigarettes for good. Among those thinking about quitting cigarettes for good, dual cigarette and e-cigarette users were also more likely (87.4 %, 95 % CI = 83.8 %, 90.2 %) than exclusive cigarette smokers (79.0 %, 95 % CI = 76.9 %, 81.0 %) to report likely quitting within the next year, whereas dual cigarette and cigar users were less likely (67.1 %, 95 % CI = 60.3 %, 73.3 %). Dual cigarette and e-cigarette users were particularly likely to report thinking about quitting all tobacco products and quitting all tobacco products within the next year.

**Table 3:** Tobacco Use Cessation Intentions Among Daily Tobacco Users (%)

	Are you thinking about quitting cigarettes for good?	Among those thinking about quitting cigarettes for good, are you likely to quit within a year? ^a^	Are you thinking about quitting all tobacco products for good? ^b^	Among those thinking about quitting all tobacco products for good, are you likely to quit within a year? ^a^
Cigarettes only	75.6 [73.7, 77.5]	79.0 [76.9, 81.0]	n/a	n/a
Cigars only	n/a	n/a	64.8 [49.9, 77.3]	69.4 [51.2, 83.0]
E-cigarettes only	n/a	n/a	77.2 [66.2, 85.4]	82.4 [72.0, 89.5]
Cigarettes + cigars	71.3 [65.9, 76.2]	67.1 [60.3, 73.3]*	64.0 [58.3, 69.4]	67.2 [60.0, 73.7]
Cigarettes + e-cigarettes	87.4 [84.0, 90.2]*	87.4 [83.8, 90.2]*	82.0 [78.1, 85.3]	87.7 [84.1, 90.6]
Cigarettes + cigars + e-cigarettes	79.8 [68.3, 87.9]	86.0 [73.9, 93.1]	72.0 [60.6, 81.2]	84.6 [71.2, 92.4]

* *p* < 0.05 compared with cigarette only user

^a^ Respondents could report that they were likely to quit smoking or the use of all tobacco products “within the next 30 days,” “within the next 6 months,” “within the year,” “longer than a year,” and “don’t know/not sure.” Respondents in the first three groups were classified as likely to quit within a year

^b^ The question on thinking of quitting use of all tobacco products was only asked of cigarette smokers who had stated that they were thinking of quitting use of cigarettes. Results in this column for categories involving cigarette smoking represent both thinking about quitting cigarettes for good and thinking about quitting all tobacco products for good

## Discussion

This study presents detailed, nationally representative estimates of dependence symptoms and cessation intentions for US adult daily cigarette, cigar, and e-cigarette users using 2012–2013 NATS data. We find that dual cigarette and cigar users report greater tobacco dependence symptoms than exclusive cigarette smokers based on higher cpd, shorter time to first tobacco use after waking, and greater likelihood of craving and withdrawal symptoms. We also find that dual cigarette and e-cigarette users are more likely to report craving and withdrawal symptoms than exclusive cigarette smokers, although they have similar cpd and time to first tobacco use. In terms of cessation intentions, dual cigarette and cigar users were less interested in quitting cigarettes than exclusive cigarette smokers, which may be indicative of greater dependence, whereas dual cigarette and e-cigarette users were more interested in quitting cigarettes than exclusive cigarette smokers.

Our finding that daily dual cigarette and cigar users may be more tobacco dependent and less likely to have cessation intentions than exclusive cigarette smokers is original and merits further investigation. Some previous studies have not found differences in cigarette dependence as measured by cpd and ttfc between exclusive cigarette and dual cigarette and cigar users [25, 26], although an analysis of nationally representative National Health and Nutrition Examination Survey data found that dual users reported smoking more cigars per day than exclusive cigar smokers [27]. It should be noted that NATS did not collect information on number of cigars smoked per day, and our analyses do not distinguish between types of cigars such as little filtered cigars, cigarillos, and large cigars, which may impact dependence symptoms. As a result, our results may underestimate dependence among users of certain cigar types.

We also find that dual cigarette and e-cigarette users are more likely to report certain tobacco dependence symptoms, such as craving and withdrawal symptoms, than exclusive cigarette smokers. These findings may suggest that these dual users are more tobacco dependent than exclusive cigarette smokers. Such an inference would be consistent with findings from observational studies that cigarette smokers who have used e-cigarettes are less likely to quit smoking than smokers who have not used e-cigarettes [25]. In our analyses, dual cigarette and e-cigarette users are also more likely to report thinking about quitting cigarettes for good than exclusive cigarette smokers. These results would be consistent with previous findings [28] that some individuals may use e-cigarettes as a means to quit smoking [29, 30], although NATS did not specifically ask about the use e-cigarettes or other tobacco products to quit smoking.

We do not find differences in time to first tobacco use between dual cigarette and e-cigarette users and exclusive cigarette smokers. One previous cross-sectional analysis found that dual cigarette and e-cigarette users were more likely to report using their first cigarette within 30 min of waking compared with exclusive cigarette smokers [31], although another analysis found no differences in ttfc between these two groups [28]. Our study also does not find a difference in cpd between exclusive daily cigarette and dual cigarette and e-cigarette users, although it is possible that some e-cigarette users reduced their tobacco consumption such that they were no longer daily users and therefore not a part of our analysis. Some researchers have suggested that some smokers may use e-cigarettes to reduce cigarette consumption [29, 30], but at the present time it remains unclear if e-cigarette use is associated with appreciable decreases in cigarette consumption. One longitudinal study found that dual cigarette and e-cigarette users were less likely than exclusive cigarette smokers to reduce cpd [32], although another analysis found that daily e-cigarette users were more likely to decrease consumption [33]. From our results, it is unclear if dual e-cigarette and cigarette users are using e-cigarettes to supplement, rather than replace, cigarette smoking, or if these dual users were more dependent and smoked more cigarettes before beginning e-cigarette use. Nevertheless, the increased cumulative tobacco exposure among these dual users may provide them with similar or higher levels of nicotine than exclusive smokers, potentially augmenting craving and withdrawal symptoms.

Our study also shows that a significant number of exclusive daily cigar and e-cigarette users report symptoms of tobacco dependence such as craving and withdrawal. The exact proportion of users reporting these symptoms varies by product category, but more than a third of exclusive daily cigar and e-cigarette users report symptoms such as strong cravings for tobacco or feeling like they really needed to use tobacco in the past 30 days.

Our results for withdrawal and craving symptoms demonstrate the value of use of a variety of dependence measures in surveys and studies. For example, we find that dual cigarette and e-cigarette users are more likely to report craving and withdrawal symptoms than exclusive cigarette smokers, although cpd and time to first tobacco use do not differ between these two groups. As a result, our assessment of general tobacco dependence symptoms revealed differences for these users that simpler measures such as cpd would have failed to capture. Our findings underscore the value of developing and using general tobacco dependence measures, including craving and withdrawal symptoms, rather than relying solely on cigarette-specific measures when analyzing dependence among users of other products.

This study does have certain limitations. Although the large sample size of the survey increases the precision of estimates, the response rate for NATS was 45 %, thus providing the potential for non-response bias. Such bias would be particularly relevant for cellular telephone-only respondents, who tend to be younger and have lower socioeconomic status than other respondents [34]. Tobacco use and dependence measures were self-reported by survey participants and therefore have the potential for misclassification or bias. NATS also did not collect information about quantity used for products other than cigarettes. The cross-sectional nature of the data does not allow us to directly assess how tobacco use patterns affect levels of tobacco dependence or cessation intentions over time. We also limited our analyses to exclusive, dual, and polyusers of cigarettes, cigars, and e-cigarettes, so our results do not include users of these products who also use smokeless tobacco, pipes, and/or waterpipes. Our analyses also focuses on current tobacco use and do not assess the effects, if any, of former tobacco use on dependence. Finally, the magnitude of differences in dependence symptoms should be considered when evaluating their impact on successful cessation outcomes.

This study provides additional information and understanding about tobacco dependence symptoms and cessation intentions among users of several commonly used tobacco products in the US. Although cigarette smoking prevalence has declined over time, tobacco use remains a leading cause of preventable death and disease in the US [35], and the use of some non-cigarette tobacco products such as e-cigarettes has increased in recent years [36]. Moreover, polytobacco use has become increasingly common among some tobacco user groups such as young adults [1]. The Family Smoking Prevention and Tobacco Control Act mandates that FDA regulates tobacco products through a standard based on population health that weighs potential benefits and harm to current, former, and never users of tobacco products. Our results indicate that there are substantial differences in the prevalence of dependence symptoms and cessation intentions among users of different combinations of tobacco products such as cigarettes, e-cigarettes, and cigars. These findings suggest that tobacco dependence among these product users is complex and multifaceted, especially in the context of dual and polytobacco use. Our results thus specifically help inform tobacco regulatory efforts aimed at protecting the health of the US population while also providing insight into dependence symptoms and cessation intentions among tobacco users more generally.

## Conclusion

This study presents important information about dependence symptoms and cessation intentions among US adult daily cigarette, cigar, and e-cigarette users that increases our understanding of dependence among tobacco users. We find that dual users of cigarettes and cigars are more likely to report greater tobacco dependence than exclusive cigarette smokers as measured by cpd and time to first tobacco use and that dual cigarette and cigar users and dual cigarette and e-cigarette users are more likely to report withdrawal and craving symptoms than exclusive cigarette smokers. We also find that many users in all product use categories, including exclusive users of cigars and e-cigarettes, exhibit symptoms of tobacco dependence. These findings have the potential to inform future research and tobacco efforts generally, given that tobacco use and dependence remain significant global public health issues, while being of particular relevance to the US specifically, given that the FDA has extended its authority to regulate all tobacco products including e-cigarettes and cigars.

## Abbreviations

CI: Confidence interval
cpd: Cigarettes per day
FDA: Food and Drug Administration
NATS: National Adult Tobacco Survey
ttfc: Time to first cigarette
US: United States

## References

[1] Fix BV, O’Connor RJ, Vogl L, Smith D, Bansal-Travers M, Conway KP, Ambrose B, Yang L, Hyland A (2014). Patterns and correlates of polytobacco use in the United States over a decade: NSDUH 2002-2011. Addict Behav.

[2] Richardson A, Williams V, Rath J, Villanti AC, Vallone D (2014). The next generation of users: prevalence and longitudinal patterns of tobacco use among US young adults. Am J Public Health.

[3] Lee YO, Hebert CJ, Nonnemaker JM, Kim AE (2014). Multiple tobacco product use among adults in the United States: cigarettes, cigars, electronic cigarettes, hookah, smokeless tobacco, and snus. Prev Med.

[4] Heatherton TF, Kozlowski LT, Frecker RC, Fagerstrom KO (1991). The Fagerstrom Test for Nicotine Dependence: a revision of the Fagerstrom Tolerance Questionnaire. Br J Addict.

[5] Fagerstrom K (2012). Determinants of tobacco use and renaming the FTND to the Fagerstrom Test for Cigarette Dependence. Nicotine Tob Res.

[6] Caraballo RS, Novak SP, Asman K (2009). Linking quantity and frequency profiles of cigarette smoking to the presence of nicotine dependence symptoms among adolescent smokers: findings from the 2004 National Youth Tobacco Survey. Nicotine Tob Res.

[7] Hu MC, Davies M, Kandel DB (2006). Epidemiology and correlates of daily smoking and nicotine dependence among young adults in the United States. Am J Public Health.

[8] Breslau N, Johnson EO, Hiripi E, Kessler R (2001). Nicotine dependence in the United States: prevalence, trends, and smoking persistence. Arch Gen Psychiatry.

[9] Kandel DB, Chen K (2000). Extent of smoking and nicotine dependence in the United States: 1991-1993. Nicotine Tob Res.

[10] Heatherton TF, Kozlowski LT, Frecker RC, Rickert W, Robinson J (1989). Measuring the heaviness of smoking: using self-reported time to the first cigarette of the day and number of cigarettes smoked per day. Br J Addict.

[11] Kozlowski LT, Porter CQ, Orleans CT, Pope MA, Heatherton T (1994). Predicting smoking cessation with self-reported measures of nicotine dependence: FTQ, FTND, and HSI. Drug Alcohol Depend.

[12] Baker TB, Piper ME, McCarthy DE, Bolt DM, Smith SS, Kim SY, Colby S, Conti D, Giovino GA, Hatsukami D (2007). Time to first cigarette in the morning as an index of ability to quit smoking: implications for nicotine dependence. Nicotine Tob Res.

[13] Piper ME, Piasecki TM, Federman EB, Bolt DM, Smith SS, Fiore MC, Baker TB (2004). A multiple motives approach to tobacco dependence: the Wisconsin Inventory of Smoking Dependence Motives (WISDM-68). J Consult Clin Psychol.

[14] Fagerstrom K, Eissenberg T (2012). Dependence on tobacco and nicotine products: a case for product-specific assessment. Nicotine Tob Res.

[15] Apelberg BJ, Corey CG, Hoffman AC, Schroeder MJ, Husten CG, Caraballo RS, Backinger CL (2014). Symptoms of tobacco dependence among middle and high school tobacco users: results from the 2012 National Youth Tobacco Survey. Am J Prev Med.

[16] Strong DR, Messer K, Hartman SJ, Conway KP, Hoffman AC, Pharris-Ciurej N, White M, Green VR, Compton WM, Pierce J (2015). Measurement of multiple nicotine dependence domains among cigarette, non-cigarette and poly-tobacco users: Insights from item response theory. Drug Alcohol Depend.

[17] Foulds J, Veldheer S, Yingst J, Hrabovsky S, Wilson SJ, Nichols TT, Eissenberg T (2015). Development of a questionnaire for assessing dependence on electronic cigarettes among a large sample of ex-smoking E-cigarette users. Nicotine Tob Res.

[18] Federal Register. Deeming Tobacco Products To Be Subject to the Federal Food, Drug, and Cosmetic Act, as Amended by the Family Smoking Prevention and Tobacco Control Act. 10 May 2016. 27192730

[19] Centers for Disease Control and Prevention (2015). National Adult Tobacco Survey.

[20] Centers for Disease Control and Prevention (2015). 2012-2013 National Adult Tobacco Survey Sample Design and Methodology Summary.

[21] Wellman RJ, DiFranza JR, Savageau JA, Godiwala S, Friedman K, Hazelton J (2005). Measuring adults’ loss of autonomy over nicotine use: the Hooked on Nicotine Checklist. Nicotine Tob Res.

[22] SAS Institute. SAS. [Version 9.4]. Cary, NC; 2012.

[23] Research Triange International. SUDAAN. [Version 11.0.1]. Research Triange Park, NC; 2015.

[24] Agaku IT, King BA, Husten CG, Bunnell R, Ambrose BK, Hu SS, Holder-Hayes E, Day HR (2014). Tobacco product use among adults--United States, 2012-2013. MMWR Morb Mortal Wkly Rep.

[25] Cohn A, Cobb CO, Niaura RS, Richardson A. The other combustible products: prevalence and correlates of little cigar/cigarillo use among cigarette smokers. Nicotine Tob Res. 2015;17(12):1473–81. 10.1093/ntr/ntv022 25634932

[26] Richardson A, Xiao H, Vallone DM (2012). Primary and dual users of cigars and cigarettes: profiles, tobacco use patterns and relevance to policy. Nicotine Tob Res.

[27] Chen J, Ketterman A, Rostron B, Day H (2014). Biomarkers of exposure among US cigar smokers: an analysis of 1999-2012 National Health and Nutrition Examinatin Survey (NHANES) data. Cancer Epidemiol. Biomarkers Prev..

[28] Rutten LJ, Blake KD, Agunwamba AA, Grana RA, Wilson PM, Ebbert JO, Okamoto J, Leischow SJ. Use of e-cigarettes among current smokers: associations among reasons for use, quit intentions, and current tobacco use. Nicotine Tob Res. 2015;17(10):1228–34. 10.1093/ntr/ntv003 PMC4592339 25589678

[29] Etter JF (2010). Electronic cigarettes: a survey of users. BMC Public Health.

[30] McRobbie H, Bullen C, Hartmann-Boyce J, Hajek P (2014). Electronic cigarettes for smoking cessation and reduction. Cochrane Database Syst Rev.

[31] Grana RA, Popova L, Ling PM (2014). A longitudinal analysis of electronic cigarette use and smoking cessation. JAMA Intern Med.

[32] Al-Delaimy WK, Myers MG, Leas EC, Strong DR, Hofstetter CR. E-cigarette use in the past and quitting behavior in the future: a population-based study. Am J Public Health. 2015;105(6):1213–9. 10.2105/AJPH.2014.302482 PMC4431097 25880947

[33] Brose LS, Hitchman SC, Brown J, West R, McNeill A. Is the use of electronic cigarettes while smoking associated with smoking cessation attempts, cessation and reduced cigarette consumption? A survey with a 1-year follow-up. Addiction. 2015;110(7):1160–8. 10.1111/add.12917 PMC4862028 25900312

[34] Blumberg SJ, Luke JV (2009). Reevaluating the need for concern regarding noncoverage bias in landline surveys. Am J Public Health.

[35] Services UDoHaH. The Health Consequences of Smoking - 50 Years of Progress: A Report of the Surgeon General, 2014. Washington: 2014.

[36] Schoenborn CA, Gindi RM. Electronic Cigarette Use Among Adults: United States, 2014. NCHS Data Brief. 2015;(217). 26555932

